# Supplementary material for: A Systematic Review of the Use of Circulating Cell-Free DNA Dynamics to Monitor Response to Treatment in Metastatic Breast Cancer Patients
Source: Cancers (Basel). 2021 Apr 10;13(8):1811. doi: 10.3390/cancers13081811 (PMC8069506; doi:10.3390/cancers13081811)
Supplement: Supplementary file 1 [file cancers-13-01811-s001.pdf]

Supplementary methods S1 Literature search

Embase.com

('circulating tumor DNA'/de OR 'cell free nucleic acid'/de OR 'methylation'/de OR (cfDNA OR ctDNA OR ccfDNA OR ((cell-free OR circulat* OR tumor-derived* OR tumour-derived*) NEAR/6 (DNA OR DNAs OR nucleic-acid*)) OR (methylat* NEAR/3 (pattern OR DNA OR DNAs OR nucleic-acid* OR gene*)) OR copy-number*):ab,ti,kw) AND ('metastasis'/exp OR 'advanced cancer'/de OR (metas* OR advanced):ab,ti,kw) AND ('breast tumor'/de OR 'breast cancer'/exp OR (((breast* OR mamma*) AND (adenocarcinoma* OR cancer* OR carcino* OR neoplas* OR tumor* OR tumour*))):ab,ti,kw) AND ('therapy'/de OR 'therapy':lnk OR 'cancer therapy'/de OR 'cancer chemotherapy'/de OR 'induction chemotherapy'/de OR 'maintenance chemotherapy'/de OR 'multimodal chemotherapy'/de OR 'cancer gene therapy'/de OR 'cancer hormone therapy'/de OR 'cancer immunotherapy'/exp OR 'cancer palliative therapy'/de OR 'multimodality cancer therapy'/de OR 'cancer combination chemotherapy'/de OR (therap* OR treatment* OR palliat* OR chemotherap*):ab,ti,kw) AND ('prediction'/de OR 'predictive validity'/de OR 'predictive value'/de OR 'monitoring'/de OR 'patient monitoring'/de OR 'drug monitoring'/de OR 'treatment outcome'/de OR 'clinical outcome'/de OR 'disease free interval'/de OR 'tumor volume'/exp OR 'treatment response'/de OR 'follow up'/de OR (predict* OR monitor* OR ((tumor* OR tumour*) NEAR/3 (burden*)) OR ((therap* OR treat* OR clinical*) NEAR/3 (outcome*)) OR follow-up OR followup):ab,ti,kw) NOT ([Conference Abstract]/lim AND [1800-2017]/py) NOT ((animal/exp OR animal*:de OR nonhuman/de) NOT ('human'/exp))

Medline (Ovid)

(Circulating Tumor DNA/ OR Cell-Free Nucleic Acids/ OR exp Methylation/ OR (cfDNA OR ctDNA OR ccfDNA OR ((cell-free OR circulat* OR tumor-derived* OR tumour-derived*) ADJ6 (DNA OR DNAs OR nucleic-acid*)) OR (methylat* ADJ3 (pattern OR DNA OR DNAs OR nucleic-acid* OR gene*)) OR copy-number*).ab,ti,kf.) AND (exp Neoplasm Metastasis/ OR (metas* OR advanced).ab,ti,kf.) AND (exp Breast Neoplasms/ OR (((breast* OR mamma*) AND (adenocarcinoma* OR cancer* OR carcino* OR neoplas* OR tumor* OR tumour*))).ab,ti,kf.) AND (therapy.fs. OR Induction Chemotherapy/ OR Maintenance

Chemotherapy/ OR Antineoplastic Combined Chemotherapy Protocols/ OR Genetic Therapy/ OR exp Immunotherapy/ OR (therap* OR treatment* OR palliat* OR chemotherap*).ab,ti,kf.) AND (Predictive Value of Tests/ OR Drug Monitoring/ OR Treatment Outcome/ OR Tumor Burden/ OR Follow-Up Studies/ OR (predict* OR monitor* OR ((tumor* OR tumour*) ADJ3 (burden*)) OR ((therap* OR treat* OR clinical*) ADJ3 (outcome*)) OR follow-up OR followup).ab,ti,kf.) NOT (news OR congres* OR abstract* OR book* OR chapter* OR dissertation abstract*).pt. NOT (exp animals/ NOT humans/)

Web of Science

TS=(((cfDNA OR ctDNA OR ccfDNA OR ((cell-free OR circulat* OR tumor-derived* OR tumour-derived*) NEAR/5 (DNA OR DNAs OR nucleic-acid*)) OR (methylat* NEAR/2 (pattern OR DNA OR DNAs OR nucleic-acid* OR gene*)) OR copy-number*)) AND ((metas* OR advanced)) AND ((((breast* OR mamma*) AND (adenocarcinoma* OR cancer* OR carcino* OR neoplas* OR tumor* OR tumour*)))) AND ((therap* OR treatment* OR palliat* OR chemotherap*)) AND ((predict* OR monitor* OR ((tumor* OR tumour*) NEAR/2 (burden*)) OR ((therap* OR treat* OR clinical*) NEAR/2 (outcome*)) OR follow-up OR followup)) NOT ((animal* OR rat OR rats OR mouse OR mice OR murine OR dog OR dogs OR canine OR cat OR cats OR feline OR rabbit OR cow OR cows OR bovine OR rodent* OR sheep OR ovine OR pig OR swine OR porcine OR veterinar* OR chick* OR zebrafish* OR baboon* OR nonhuman* OR primate* OR cattle* OR goose OR geese OR duck OR macaque* OR avian* OR bird* OR fish*) NOT (human* OR patient* OR women OR woman OR men OR man))) AND DT=(Article OR Review)

Cochrane Central

((cfDNA OR ctDNA OR ccfDNA OR ((cell-free OR circulat* OR tumor-derived* OR tumour-derived*) NEAR/6 (DNA OR DNAs OR nucleic-acid*)) OR (methylat* NEAR/3 (pattern OR DNA OR DNAs OR nucleic-acid* OR gene*)) OR copy-number*):ab,ti,kw) AND ((metas* OR advanced):ab,ti,kw) AND ((((breast* OR mamma*) AND (adenocarcinoma* OR cancer* OR carcino* OR neoplas* OR tumor* OR tumour*))):ab,ti,kw) AND ((therap* OR treatment* OR palliat* OR chemotherap*):ab,ti,kw) AND ((predict* OR monitor* OR ((tumor* OR tumour*)

NEAR/3 (burden*)) OR ((therap* OR treat* OR clinical*) NEAR/3 (outcome*)) OR follow-up OR followup):ab,ti,kw)

Google Scholar

cfDNA|ctDNA|"cell-free|circulating DNA|DNAs" metastasis|metastases "breast|mamma cancer|neoplasm|neoplasms|tumor|tumour" therapy|treatment|chemotherapy predict|prediction|monitor|monitoring|followup|follow-up
